# *Coxiella* Endocarditis as the Cause of Recurrent Fever and Brain Abscess in a Patient with Complex Congenital Heart Disease: A Case Report and Literature Review

**DOI:** 10.1155/2020/7894574

**Published:** 2020-06-24

**Authors:** Ioannis A. Ziogas, Alexandros P. Evangeliou, Olga Tsachouridou, Alexandra Arvanitaki, Afroditi Tsona, Vassilios Kamperidis, Marianthi Papagianni, Theofilos Panagiotidis, Ilias A. Tourtounis, George Giannakoulas, Symeon Metallidis

**Affiliations:** ^1^School of Medicine, Aristotle University of Thessaloniki, Thessaloniki, Greece; ^2^Infectious Diseases Unit, First Department of Internal Medicine, AHEPA University Hospital, Aristotle University of Thessaloniki, Thessaloniki, Greece; ^3^First Department of Cardiology, AHEPA University Hospital, Aristotle University of Thessaloniki, Thessaloniki, Greece

## Abstract

**Introduction:**

Blood culture-negative infective endocarditis (BCNIE) can present subtly and is associated with a diagnostic delay leading to increased morbidity and mortality. *Case Report*. We present the case of an 18-year-old male with a history of complex congenital heart disease and 3-year intermittent episodes of fever of unknown origin, who was referred to our hospital for upper and lower extremity focal seizures. Laboratory blood tests were normal, blood cultures were negative, and brain imaging revealed an abscess. Cardiology consultation was requested, and transthoracic echocardiography revealed an intracardiac vegetation. Empiric antibiotic treatment with sultamicillin, gentamycin, and meropenem was initiated. Serology testing was positive for *Coxiella burnetii*, and the diagnosis of BCNIE was established. The antibiotic course was changed to oral doxycycline for 36 months and led to resolution of IE, with no vegetation detected on TTE after 15 months.

**Conclusion:**

BCNIE is a life-threatening disease entity that can lead to severe complications, such as valve regurgitation, emboli, and death. Patients with congenital heart disease are particularly vulnerable to IE. Timely diagnosis and antibiotic management are of paramount importance in order to avoid the potentially fatal sequelae.

## 1. Introduction

Blood culture-negative infective endocarditis (BCNIE) refers to the cases of infective endocarditis (IE) in which usual blood culture methods fail to identify a causative microorganism, and BCNIE may account for up to 70% of all endocarditis cases [1]. IE is commonly associated with major complications, including severe valvular dysfunction (mainly regurgitation) leading to heart failure and occasionally embolic stroke. Overall prognosis can be poor due to the high risk of recurrence and increased mortality rate [2]. Brain abscess constitutes a rare complication, with an incidence of up to 6% in patients with IE irrespective of neurological status [3, 4].

BCNIE poses a significant diagnostic challenge due to the initially negative cultures, which can cause a clinically significant diagnostic delay; this may contribute to a further increase in morbidity and mortality in patients with BCNIE [5]. *Coxiella burnetii*, the causative agent of Q fever and a potential cause of BCNIE, is an obligate intracellular pathogen that cannot be isolated with the routine culture methods. Common clinical manifestations include fever, cardiac failure, splenomegaly, and hepatomegaly [6].

We aimed to present a rare clinical manifestation of BCNIE secondary to *Coxiella burnetii* that was associated with a diagnostic delay and a life-threatening complication in a young adult with a past medical history of complex congenital heart disease (CHD).

## 2. Case Report

An 18-year-old male was referred to our hospital after experiencing two episodes of left upper and lower extremity focal seizures over the past 10 days, which were managed with antiepileptic medication. His history of present illness began 15 days prior to admission when he experienced left-sided tingling sensation without any other symptoms. At that time, he visited the local emergency department, where neurology consultation was provided. Computed tomography (CT) scan of the brain showed a ring-enhancing parasagittal lesion on the right cerebral hemisphere. The patient was scheduled for brain magnetic resonance imaging (MRI) later the same month and was discharged without any further interventions.

Upon admission, we examined a 32 kg and 1.46 m young adult with a Glasgow Coma Scale of 15. From the vital sign standpoint, he was febrile (37.5°C) with an oxygen saturation of 76% at rest, while heart rate was 87 beats/minute and systolic and diastolic blood pressures were 124 mmHg and 60 mmHg, respectively. On physical examination, perioral cyanosis, digital clubbing, and lower extremity edema were appreciated. His past medical history was notable for pulmonary valve atresia with ventricular septal defect (VSD), major pulmonary collateral arteries (MAPCAs) status after five cardiothoracic operations, and head injury at the age of seven years leading to hemorrhage that was managed conservatively. He lived in the countryside, but no contact with animals was reported. Several recurrent fever episodes of unknown origin occurred during the preceding 3 years (with the last episode occurring 2 months before hospital admission), all of which were managed conservatively.

**Figure 1:**
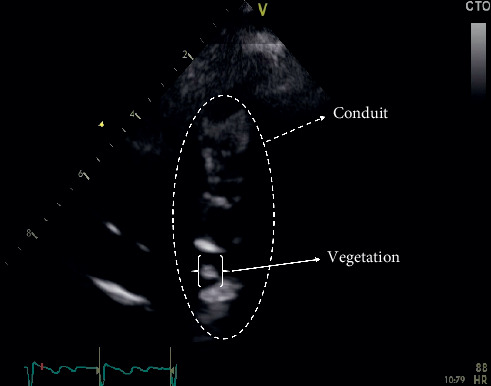
Transthoracic echocardiogram (TTE): a calcified vegetation, 4.0 mm × 3.0 mm in size, with independent mobility, was identified inside the conduit.

**Figure 2:**
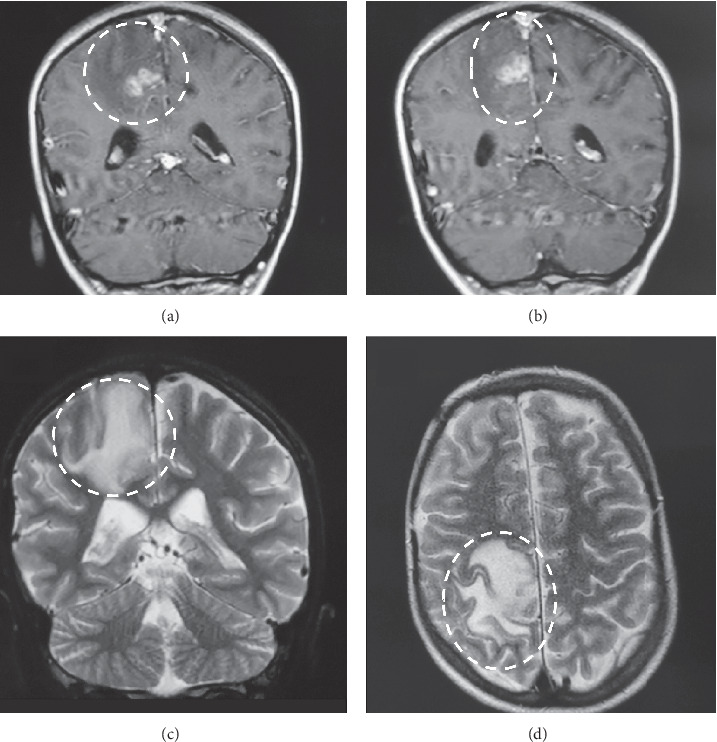
Brain magnetic resonance imaging (MRI): 2.2 × 2.0 cm ring-enhancing parasagittal lesion in the right parietal lobe.

X-rays of the head and chest did not reveal any abnormality. Complete blood count and comprehensive metabolic panel were significant for a normal white blood cell count (5.46 K/*μ*L) and hemoglobin (16.1 g/dL), but increased C-reactive protein (5.3 mg/dl), red blood cell count (9.4 × 10^12^/L), alkaline phosphatase (182 ΙU/L), blood urea nitrogen (70 mg/dL), lactose dehydrogenase (478 ΙU/L), potassium (5.2 mEq/L), d-dimers (3510 *μ*g/L), and NT-pro-B natriuretic peptide (7,312 pg/mL). The viral panel was negative for human immunodeficiency virus, hepatitis B virus, and hepatitis C virus, while blood cultures also came back negative. The patient underwent a transthoracic echocardiogram (TTE), which revealed a calcified conduit extending from the right ventricle to the pulmonary artery with severe pulmonary valve stenosis, a peak systolic gradient of 78 mmHg, a large VSD, and severe pressure overload of the right ventricle with flattening of the interventricular septum. A calcified vegetation, 4.0 mm × 3.0 mm in size, with independent mobility, was also identified inside the conduit (Figure 1 and video provided in Supplementary Materials (available here)). The right ventricle was severely hypertrophied with impaired systolic function. This pointed out our diagnostic thinking towards culture-negative endocarditis, and therefore, we ordered blood serologies for *Bartonella henselae*, which came back negative, and *Coxiella burnetii*, which were positive (IgM-phase I: 1/192 and IgG-phase II: 1/2048). Brain MRI showed a 2.2 cm × 2.0 cm heterogenous ring-enhancing parasagittal lesion in the right parietal lobe consistent with brain abscess (Figure 2). Consequently, the diagnosis of recurrent fevers secondary to brain abscess attributed to *Coxiella burnetii* culture-negative endocarditis was established.

The patient was on aspirin and furosemide at home for his corrected cyanotic CHD. Based on the lesion seen on the CT scan, the patient was started on mannitol and steroids in order to reduce the abscess-related brain edema. The brain abscess was managed conservatively as the patient was hemodynamically stable. Empirical antibiotic treatment (sultamicillin and gentamycin) was initiated, and after the diagnosis of brain abscess, from MRI findings as well as due to high antibiotic resistance, meropenem was added to the existent therapy. When *Coxiella* serologies came back positive, the antibiotic course was modified to doxycycline for a total of 36 months. Double combination therapy with bosentan and sildenafil was also initiated for the significant pulmonary hypertension documented on TTE. A right ventricular systolic pressure of 78 mmHg without significant conduit stenosis was also reported on TTE. The patient was scheduled for a repeat CT scan of the brain, as well as for a follow-up visit with a cardiologist for the assessment of endocarditis sequelae and cyanotic CHD-induced pulmonary hypertension. Fifteen months after the diagnosis of IE, TTE examination revealed no recurrent vegetation, while improvement of right ventricular contractility was also documented.

## 3. Discussion

BCNIE is defined as evidence of IE with three consecutive pairs of sterile blood cultures, with the use of standard cultural methods [7]. One positive blood culture for *Coxiella burnetii*, IgG-phase I antibody titers >1 : 800, or positive polymerase chain reaction of a surgical specimen are included in the modified criteria for BCNIE diagnosis [8, 9], but were not included in the initially published Duke criteria for the diagnosis of IE [10]. Endocarditis is a major clinical manifestation of chronic Q fever [11], which can also lead to other severe complications, such as heart valve dysfunction or systemic emboli [12, 13]. Recurrent episodes of fever or focal neurologic signs should prompt extensive workup, including brain imaging and TTE, in order to rule out brain abscess and IE. BCNIE is particularly notorious for its complications, and therefore, timely diagnosis and early initiation of antibiotic and sometimes antithrombotic treatment is of paramount importance. Indications for surgical management include failure of medical therapy, damaged heart valve, increased risk of thromboembolism or perivalvular abscess [14]. Interestingly, Kang et al. reported that early valve surgery is more effective than medical therapy with a lower risk of systemic embolization, and lower mortality rate in patients with IE and vegetations >15 mm on echocardiography [15]. Nevertheless, the treatment of choice for *Coxiella*-induced IE remains controversial. Data suggest that long-term (>18 months) antibiotic treatment with doxycycline and hydroxychloroquine, or surgical management if needed, is associated with optimal outcomes [6, 8]. In this patient, oral doxycycline alone was prescribed for a period of 36 months after discharge, and complete resolution of IE, based on TTE, was documented by 15 months.

Patients with a history of complex cyanotic CHD are particularly prone to developing IE [16]. Studies have shown that a history of CHD is reported in approximately 4%–14% of patients diagnosed with Q fever IE [17, 18]. More specifically, Q fever IE has been reported in patients with transposition of the great vessels [19], truncus arteriosus and tetralogy of Fallot [20], VSD [21, 22], dextrocardia [23], cactus aorta [24], and coarctation of the aorta [25]. Moreover, although several cases of brain abscess secondary to IE have been published to date [26–28], the present report constitutes the first case of brain abscess attributed to *Coxiella burnetii* BCNIE.

There is great variability in the overall seroprevalence of *Coxiella burnetii* in the Mediterranean region (3–38%) [29, 30]. An epidemiological study from Crete, Greece, reported that the incidence of *Coxiella burnetii* in patients with zoonoses was 13.6% and 8.6% in adults and children, respectively [31]. Additionally, Papakonstantinou et al. assessed another 82 patients diagnosed with IE in Crete, Greece. Thirteen (15%) were classified as BCNIE, and only one case was attributed to *Coxiella burnetii* [32]. In another study from Athens, Greece, Loupa and colleagues reported that, among 101 cases of IE, only one was secondary to *Coxiella burnetii* [33].

## 4. Conclusion

BCNIE should be included in the differential diagnosis of recurrent fever of unknown origin, particularly in CHD patients, due to the increased risk of IE. Focal neurological signs necessitate prompt brain imaging and cardiology consultation, while serology tests should be obtained in case of negative blood cultures.

## Abbreviations

BCNIE: Blood culture-negative infective endocarditis
IE: Infective endocarditis
CT: Computed tomography
MRI: Magnetic resonance imaging
VSD: Ventricular septal defect
MAPCAs: Major pulmonary collateral arteries
TTE: Transthoracic echocardiogram
CHD: Congenital heart disease.

## Supplementary Materials

Supplementary Materials TTE examination revealed a calcified conduit extending from the right ventricle to the pulmonary artery with severe pulmonary valve stenosis. A calcified vegetation, 4  mm × 3  mm in size, with independent mobility, was reported inside the conduit.
